# 2018 Zika Health Brigade: Delivering Critical Health Screening in the U.S. Virgin Islands

**DOI:** 10.3390/tropicalmed5040168

**Published:** 2020-11-09

**Authors:** Shana Godfred-Cato, S. Nicole Fehrenbach, Megan R. Reynolds, Romeo R. Galang, Dan Schoelles, Lessely Brown-Shuler, Braeanna Hillman, Leah DeWilde, Andra Prosper, Amy Hudson, Cynthia A. Moore, Esther M. Ellis

**Affiliations:** 1 Centers for Disease Control and Prevention, National Center on Birth Defects and Developmental Disabilities, Division of Birth Defects and Infant Disorders, Atlanta, GA 30341, USA; ekk5@cdc.gov (S.N.F.); xah6@cdc.gov (M.R.R.); cam0@cdc.gov (C.A.M.); 2 Centers for Disease Control and Prevention, National Center for Chronic Disease Prevention and Health Promotion, Division of Reproductive Health, Atlanta, GA 30341, USA; ydh0@cdc.gov; 3 Deloitte Consulting LLP, New York, NY 10112, USA; dschoelles@deloitte.com; 4 Chickasaw Nation Industries, Norman, OK 73071, USA; lessely.brownshuler@chickasaw.com; 5 U.S. Virgin Islands Department of Health, Christiansted, VI 00820, USA; braeanna.hillman@doh.vi.gov (B.H.); Leah.Dewilde@doh.vi.gov (L.D.); andra.prosper@doh.vi.gov (A.P.); esther.ellis@doh.vi.gov (E.M.E.); 6 U.S. Virgin Islands Department of Health, Charlotte Amalie, VI 00802, USA; 7 Eagle Global Scientific, LLC, San Antonio, TX 78248, USA; nqz0@cdc.gov

**Keywords:** health brigade, congenital Zika syndrome, congenital Zika infection, health screenings

## Abstract

In 2017, Hurricanes Irma and Maria caused significant damage to the United States Virgin Islands (USVI), heightening the challenges many residents faced in accessing adequate healthcare and receiving recommended Zika virus screening services. To address this challenge, the USVI Department of Health (DOH) requested technical assistance from the Centers for Disease Control and Prevention (CDC), the Health Resources and Services Administration (HRSA), and the American Academy of Pediatrics (AAP) to organize a health brigade to bring needed medical care to an underserved population. It also established the development of important partnerships between federal and private partners as well as between clinical providers and public health entities such as the Epidemiology & Disease Reporting, Maternal Child Health (MCH), and Infant and Toddlers Programs within the DOH, and local clinicians. This health brigade model could be replicated to ensure recommended evaluations are delivered to populations that may have unmet medical needs due to the complexity of the conditions and/or rural location.

## 1. Introduction

Zika virus was first identified in 1947 in a rhesus monkey in Uganda. A link between Zika virus infection and microcephaly was reported in October 2015 in Brazil, and by February 2016, the World Health Organization (WHO) declared that Zika virus infection associated with microcephaly constituted a Public Health Emergency of International Concern (PHEIC) [1]. Exposure to Zika virus infection during pregnancy can cause serious defects of the brain and eye and has been associated with neurodevelopmental abnormalities, such as seizures, joint contractures, swallowing difficulties, vision impairments, and hearing loss, in infants [2,3].

Since the discovery of the virus, over 37,000 people have tested positive for Zika virus in the US territories, 3930 of which were pregnant women [4]. In the United States Virgin Islands (USVI), approximately 290 infants have been born to mothers with confirmed or probable Zika virus infection during pregnancy [5].

In 2017, Hurricanes Irma and Maria caused significant damage to USVI, heightening the challenges many residents faced in accessing adequate healthcare and receiving recommended Zika virus screening services. Pediatric specialty care in USVI is typically available through traveling providers, coordinated through the USVI DOH and MCH offices, or adult providers who see children intermittently. Patients also travel to see pediatric specialists outside the territory. As of January 2016, one pediatric neurologist and one developmental pediatrician provided services to USVI; both traveled from outside the territory. There were no pediatric ophthalmologists or audiologists serving the territory.

Due to the lack of access to pediatric specialty services to evaluate children born to mothers with confirmed or probable Zika virus infection during pregnancy in USVI after the hurricanes, the USVI DOH contacted the CDC for assistance with bringing pediatric specialty services to the territory to evaluate children born to mothers with confirmed or probable Zika virus infection during pregnancy in alignment with the clinical guidance [6]. A health brigade model was determined to be an effective means of bringing pediatric specialists to provide screening services. This model has been used in many countries for decades to temporarily bring healthcare providers and in some cases prolonged medical care to those with lack of access. [7,8] The pediatric specialists provided age-appropriate screenings (i.e., neurological, vision, developmental, and hearing) consistent with the Centers for Disease Control and Prevention’s (CDC) interim guidance on the diagnosis, evaluation, and management of infants exposed to Zika virus infection during pregnancy [6].

In March 2018, the USVI DOH, with support from CDC, the Health Resources and Services Administration (HRSA), and the American Academy of Pediatrics (AAP), conducted a health brigade to deliver these screenings. HRSA and USVI DOH are continuing to collaborate on ways to sustainably bring pediatric specialty providers to the islands intermittently to continue follow-up care.

## 2. Materials and Methods

Working with the AAP, the CDC recruited pediatric specialty providers to conduct recommended follow-up screening for children born to mothers with confirmed or probable Zika virus infection during pregnancy. Pediatric specialists included neurologists, audiologists, ophthalmologists, and developmental pediatricians, as well as child life specialists.

### 2.1. Recruitment of Families

The USVI DOH tracks cases of pregnant women testing positive for Zika virus and their children and provided initial contact information for outreach to these women. To broadly reach families who may have been impacted by Zika virus infection during pregnancy, communication techniques such as radio and social media advertisements, flyers, and provider engagement were used to disseminate information about the health brigade. These communication techniques also provided instructions about how to schedule appointments for the free and voluntary evaluations and screenings.

Provider outreach consisted of USVI and CDC staff visiting local pediatric offices and sharing printed flyers about the health brigade. The flyer included a toll-free number and dates and locations of the brigade, as well as a description of the pediatric screenings that would be conducted. Local pediatric providers were encouraged to inform their patients of the upcoming event, share flyers with the families, and encourage them to attend with their children.

### 2.2. Recruitment of Providers

Based on the clinical guidance for infants born to mothers with confirmed or probable Zika virus during pregnancy, pediatric specialists were recruited to provide the recommended evaluations and screenings [6,9,10,11]. The AAP and HRSA recommended specific pediatric specialty providers with experience working with infants affected by Zika virus and delivering health education to families. Providers traveled from across the United States and volunteered their time to participate in the health brigade.

Recruitment efforts took into consideration many conflicting schedules when planning the health brigade. To address these challenges, recruitment for physicians was initiated 90 days prior to the health brigade to allow enough time to adjust clinic schedules and staffing. Additionally, providers could participate in the health brigade in part or in its entirety based on their availability.

Providers were required to hold a USVI institutional medical license to practice in the territory. The medical licenses were processed using an emergency use mechanism and were valid for the duration of the health brigade. The home institutions for the specialty providers allowed continued medical malpractice insurance coverage for this time period.

Two certified child life specialists participated in the health brigade to provide emotional support to the children and their families. Activities were developed to help the infants and their families cope with the stress and anxiety of the screenings.

Local providers in the territory caring for these infants were invited to participate in an educational session on pediatric neurologic examinations. As an incentive to participate, local physicians and nurses were offered continued education (CE) credit for attending the lecture. The providers were also encouraged to participate in the health brigade screenings for their patients. 

### 2.3. Clinical Model

The health brigade was held in the MCH clinics on both St. Thomas and St. Croix. During the planning phase, DOH and CDC staff toured the clinic facilities, reviewed the functional layout to inform the health brigade set-up, and photographed existing supplies and equipment to create an itemized inventory. The MCH medical buildings included examination rooms, medical equipment, and administration space. Pediatric specialty providers remotely reviewed the equipment inventory and identified additional critical specialty equipment required to complete the anticipated screenings, such as ophthalmology and audiology equipment [10,11]. Pediatric specialty providers, the CDC, and partner organizations contributed equipment. The CDC provided financial support through a cooperative agreement and contractual support.

On patient arrival to the health brigade, a nursing assistant was available to help families complete the consent form and additional patient forms (health history form, developmental screening forms) and to take growth parameter measurements. In order to ensure appropriate techniques were used, a refresher training for growth parameter measurements was given prior to the brigade [12]. Support staff assisted with translation for families who spoke Spanish or Haitian Creole. A structured step-by-step process was developed to guide families through the health brigade’s activities, from intake to screening completion. The process included initial intake, developmental assessment using Ages & Stages Questionnaires, Third Edition (ASQ-3), Ages & Stages Questionnaires: Social-Emotional, Second Edition (ASQ:SE-2), and the Modified Checklist for Autism in Toddlers, Revised (M-CHAT-R), neurodevelopmental screening performed by neurologists, audiological screening using auditory brainstem response (ABR) and otoacoustic emissions (OAE), behavioral audiometry evaluation depending on the patient’s age and level of cooperation, and an ophthalmologic eye examination to assess ocular structure and visual function. Each child was seen by all specialties in varying order depending on the physical layout of each clinic. As families proceeded through the clinic, they received support from nursing assistants, nurses, child life specialists, and other DOH volunteers.

As part of checkout, to evaluate parents’ satisfaction with the health brigade, a survey was given about the health brigade clinical experiences, including scheduling, participating, and clinical care and about parental interactions with the child life specialists who assisted with siblings and comforted parents and patients throughout the long clinic day. Each chart was reviewed for medical record completeness and to ensure all needed examinations were performed prior to the families leaving the clinic. The families received a summary of their visit (including examinations performed and any specialty or recommended follow-up referrals). Families also received resources and tools from the “Learn the Signs. Act Early.” program so parents can participate in tracking developmental milestones of their infant [13].

## 3. Results

Four staff conducted over 500 phone calls over a two-month time period prior to the brigade and connected with 148 families with children born to mothers exposed to Zika virus during pregnancy who were at the greatest risk for adverse outcomes. Thirteen pediatric specialty providers traveled to USVI and worked with 17 CDC, DOH, and MCH staff for a total of 30 personnel working 4200 h in the planning and execution of the Zika health brigade.

Recommended screenings were performed for 88 children born to mothers exposed to Zika virus during pregnancy [14]. Follow-up services were recommended for 62 patients, including services to pediatric audiology, neurology, ophthalmology, developmental pediatrics, early intervention services, or the Infant and Toddlers Program [14]. Thirty patients were referred to more than one follow-up service.

As part of their feedback, parents responded overall that they felt the health brigade was useful. Of note, 97% of parents shared that they valued working with the child life specialists, in particular, who provided support and distraction for the patients during the examinations and support for siblings and parents during the clinic day.

## 4. Discussion

The successful execution of this health brigade emphasized the importance of improved access to pediatric specialty providers and provision of recommended screenings for infants born to mothers with confirmed or probable Zika virus infection during pregnancy.

There are several important practical lessoned learned from this experience. First, existing healthcare facilities, in this case the MCH clinics, were an appropriate clinic space to modify to host a health brigade. Adequate space was essential for some specialty procedures, in particular for the audiologists who required the use of an audiometric booth to perform the hearing screenings for children of various ages.

Second, planning ahead was critical to allow adequate time to work through complex logistics, recruitment of specialty and local providers and staff, and promotion of the event throughout the community.

Third, because of the breadth of services provided, an efficient clinical flow was necessary for patients to receive all services in a timely manner. A streamlined consent form ensured check-in ran smoothly. It was important to identify specialty exams that required a longer length of time so that schedules could account for those times to avoid delays during the screenings.

Fourth, adequate translation services were needed for patients who spoke a variety of languages. Despite having Spanish and Haitian Creole translators available, we found there were more patients who needed translation services than the translators’ time could accommodate.

Fifth, there were many challenges with communication. Multiple outreach efforts were necessary for recruitment. As noted, many families heard about the health brigade from their primary care provider and were more likely to attend if the invitation came from a trusted source. It was important, during this clinic, to clearly communicate recommended follow-ups and referrals directly to the families. Furthermore, the development of a clear communication channel between health brigade specialty providers and subsequent care providers was essential to convey the need for recommended follow-ups.

The CDC and USVI continue to collaborate to follow up children born to mothers with confirmed or probable Zika virus infection during pregnancy. HRSA and USVI DOH are developing a sustainable model to bring pediatric specialty providers to USVI periodically to continue to screen the patients seen during the health brigades as well as those that were unable to attend and met the criteria for inclusion. One of the pediatric specialists that attended the health brigade now returns quarterly to continue follow-up care of the infants. The staff education given during the brigade planning phase such as head circumference measurement refreshers and neurodevelopmental form training continues to be used in the two clinics.

Telemedicine has been explored as a potential solution for providing access to specialty pediatric care but has not been implemented. Many of the screenings require close coordination and planning because the evaluations are intensively hands-on, e.g., ophthalmologic examination. Furthermore, the telecommunications infrastructure within USVI is unstable, especially following natural disasters like hurricanes when communication via phone and internet may be unavailable.

## 5. Conclusions

The health brigade model is a useful tool for any disaster-affected region or underserved areas with limited access to adequate healthcare [15]. The relationships fostered during this health brigade may improve future relationships and communication between stakeholders to improve care for infants exposed to Zika virus during pregnancy and other children with special healthcare needs. Better stakeholder communication may ultimately improve access to care for all children in the territory. This approach can be used in the future for patients with either specific medical conditions with limited access to specialty screenings or for patients with limited access to care due to disaster or rural conditions.
